# Clinical efficacy of CT-guided ^125^I brachytherapy in patients with local residual or recurrent hepatocellular carcinoma after thermal ablation

**DOI:** 10.1186/s13244-022-01327-z

**Published:** 2022-12-06

**Authors:** Wenliang Zhu, Zhihui Zhong, Huzheng Yan, Huanqing Guo, Meigui Xiao, Xu He, Fei Gao, Fujun Zhang

**Affiliations:** 1 Department of Minimally Invasive and Interventional Radiology, State Key Laboratory of Oncology in South China, Collaborative Innovation Center for Cancer Medicine, Sun Yat-Sen University Cancer Center, 651 Dongfeng Road, East, Guangzhou, 510060 People’s Republic of China; 2 Department of Interventional Radiology, The Third Affiliated Hospital, Sun Yat-Sen University, No. 600, Tianhe Road, Tianhe District, Guangzhou, 510630 People’s Republic of China; 3 ZhuHai Interventional Medical Center, ZhuHai People’s Hospital (ZhuHai Hospital Affiliated With Jinan University), Jinan University, ZhuHai, 519000 Guangdong People’s Republic of China

**Keywords:** ^125^I brachytherapy, Hepatocellular carcinoma, Local residual or recurrent, Thermal ablation

## Abstract

**Objectives:**

Treatment methods of local residual or recurrent hepatocellular carcinoma (HCC) after thermal ablation are limited. Therefore, our study aimed to explore the efficacy and prognostic factors of ^125^I brachytherapy for local residual or recurrent lesion after thermal ablation.

**Methods:**

A total of 114 patients with 212 local residual or recurrent HCC tumors after thermal ablation underwent ^125^I brachytherapy. Local progression-free survival (LPFS) and prognostic factors were analyzed by Kaplan–Meier curves and the Cox model.

**Results:**

After a 6-month follow-up, the percentage of patients who achieved complete response (CR), partial response (PR), and stable disease (SD) was 57%, 13.2%, and 5.2%, respectively. The 1-, 2-, and 3-year LPFS rates were 58.7%, 50.0%, and 41.2%, respectively. Portal vein tumor thrombus (PVTT) (*p* = 0.03), the number of intrahepatic tumors (*p* = 0.01), and AFP level (*p* = 0.02) were independent risk factors for local tumor progression (LTP). The median LPFS in patients without PVTT (22 months) was much longer compared to those with PVTT (10 months). The median LPFS in patients with less than three intrahepatic lesions improved from 17 to 24 months. The median LPFS was only 5 months in the high AFP group, but was prolonged with a decrease in AFP level (24 months). No severe complications were recorded. All complications were controllable and treatable.

**Conclusions:**

CT-guided ^125^I brachytherapy was a safe and effective treatment for patients with local residual or recurrent HCC after thermal ablation to improve local control rate.

## Key points

- ^125^I brachytherapy is a treatment for locally recurrent HCC after ablation.
- ^125^I brachytherapy combined with ablation may improve tumor control.
- Vascular invasion, multiple lesions, and AFP are prognostic risk factors.
- ^125^I brachytherapy is a safe method with a curative effect.

## Introduction

According to the International Agency for Research on Cancer [1], the incidence of liver cancer accounts for 4.7% of the global tumor incidence and is ranked sixth. However, the mortality rate is twice the incidence rate and ranked third in 2020, indicating that the treatment is challenging and the treatment model needs to be improved to reduce the mortality rate further. Hepatocellular carcinoma (HCC) is the main tissue type of primary liver cancer, accounting for more than 90%, and curative therapies such as surgical resection, liver transplant, and ablative techniques offer the chance of long-term response and improved survival. However, about 40% of HCC patients are locally advanced at the time of diagnosis (stage IIb/IIIa according to the Chinese liver cancer staging standard) [2]. Therefore, according to the European and American Liver Cancer Association standards [3], these patients have lost the opportunity for surgical resection. However, in China, patients with locally advanced HCC can undergo surgical resection if certain conditions are met. Unfortunately, the 5-year recurrence rate is over 70% after surgical resection. Furthermore, 55% of HCC patients who underwent surgical resection plus postoperative adjuvant therapy had tumor recurrence at a median of 22 months postoperatively [4, 5].

Local thermal ablation therapy, including radiofrequency ablation (RFA) or microwave ablation (MWA), has been widely used in recent years and provides curative results for patients with early-stage liver cancer [6–8]. However, for tumors near large blood vessels (e.g., the hepatic vein or inferior vena cava), the diaphragm, abdominal organs (gallbladder and gastrointestinal tissues), or the pericardium, thermal ablation may result in thermal damage to these tissues and a higher recurrence rate [9–11]. In addition, the size > 3 cm and a location immediately adjacent to the regions mentioned above lead to a significantly lower rate of tumor ablation, which is prone to local residual or recurrent tumors [12]. For tumor recurrent or residual tumors with the above characteristics, local treatments, such as repeated ablation or stereotactic body radiation therapy (SBRT), are used clinically [11, 13].

^125^I brachytherapy could inhibit tumor progression through continuously emitting low doses of X-rays and γ-rays, with the half-life dose reaching 140–160 Gy. ^125^I brachytherapy has been widely used in the treatment of various solid malignant tumors such as prostate cancer, lung cancer, liver cancer, pancreatic cancer, and metastatic tumors [14–17]. The advantages of ^125^I brachytherapy include local high dose within tumor, less radiation damage to normal tissue around the tumor, and no respiratory movement effect [18]. CT-guided ^125^I brachytherapy is safe to HCC adjacent to the subcapsular, large blood vessels, gallbladder, or subdiaphragm. Previous studies showed that ^125^I brachytherapy yielded good clinical efficacy and safety in patients with HCC [19]. Thus, the CT-guided ^125^I brachytherapy was applied to the local recurrent or residual tumor after thermal ablation in our center. To evaluate the efficacy and safety of CT-guided ^125^I brachytherapy, we retrospectively studied 114 patients with local residual or recurrent HCC after thermal ablation.

## Materials and methods

### Patient selection

The Institutional Review Board approved this study. HCC was diagnosed according to the practice guidelines of the American Association for the Study of Liver Diseases (AASLD) [3], and the stage was according to the Barcelona Liver Clinic (BCLC) stage [20].

The inclusion criteria were as follows: 1. The patient was diagnosed with hepatocellular carcinoma; 2. the patient had a Child–Pugh classification of grade A or grade B and an ECOG score ≤ 2; and 3. the patient had an estimated survival time of more than 3 months. The exclusion criteria were as follows: 1. patients with no indication for surgery such as coagulation dysfunction, organ failure, intolerable surgery, or complicated by severe infection and 2. patients lacking procedural information. Between April 2010 and May 2021, 126 consecutive patients were included in this retrospective study. Five patients refused or were considered unsuitable for the ^125^I brachytherapy procedure, and seven patients lacking procedural information were excluded. A total of 114 patients (93 men and 21 women; mean age 58 years, range 26–84) with 212 tumors who underwent CT-guided ^125^I brachytherapy procedure for local residual or recurrent HCC after thermal ablation and fulfilled the inclusion criteria were enrolled in this study.

### CT-guided ^125^I brachytherapy procedure

^125^I seed is a synthetic anti-tumor seed with a length of 4.5 mm and a diameter of 0.8 mm. The shell of the seed is made of titanium and contains silver column with liquid ^125^I isotopes adsorbed; ^125^I seed inhibits tumor progression by emitting low-energy X- and γ-rays. The seed activity chosen was 0.8 mCi. The ray energy is 27–32 keV, the half-life is 59.6 days, the tissue semivalent layer is 2 cm, and the lead semivalent layer is 0.025 mm.

**Fig. 1:**
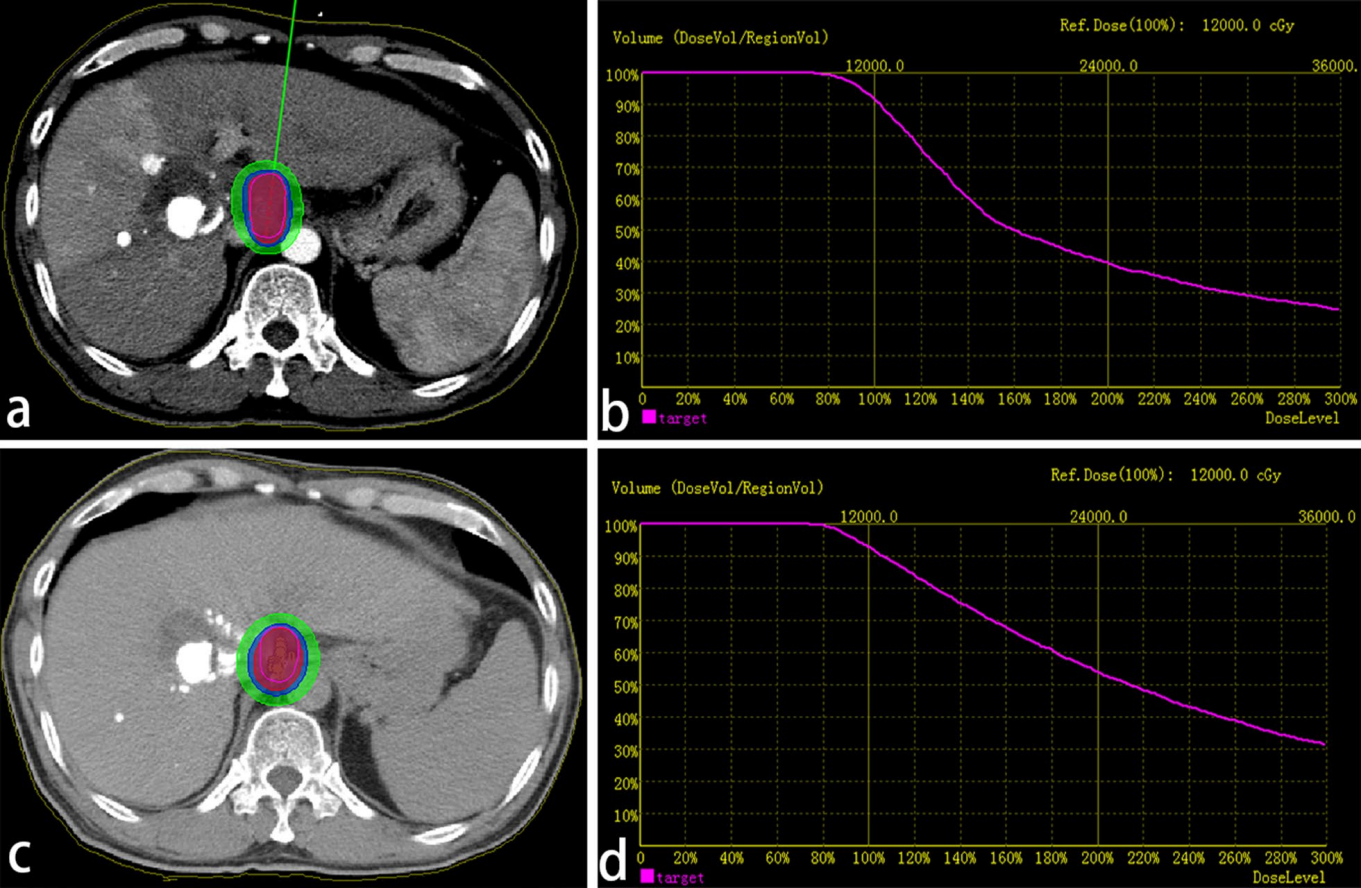
**a** Preoperative TPS. The green straight line and the red area represent the puncture path and the gross tumor outline, respectively. **b** Preoperative dose-volume histograms (DVH). The prescribed dose (PD) was 120 Gy. 90% of the tumor volume received 123 Gy (D90 = 123 Gy), and 91.7% of the tumor target received 100% of the prescribed dose (V100 = 92.5%). **c** Postoperative practical radiation dose distribution. **d** Postoperative DVH, D90 = 127.5 Gy, V100 = 92.8%. The postoperative practical radiation dose almost matches the preoperative radiation dose

The CT-guided ^125^I brachytherapy procedure was described in our previously published literature [21]. Based on the preoperative enhanced computerized tomography (CT) or magnetic resonance imaging (MRI), clinical target volume (CTV), planned target volume (PTV), and puncture path were delineated by the physicist (Fig. 1). In addition, the mean prescribed radiation dose (120, 110–140 Gy), the required number of ^125^I seeds, seed distribution, and dose-volume histogram (DVH) were generated by the treatment planning system (TPS) (RT-RSI, Beijing Atom and High Technique Industries Inc., Beijing, China). According to PTV, the dose could achieve at least 95% of the prescribed dose (Vl00 > 95%).

For the purpose of convenient operation and avoidance of vessel injuries, the patient’s body position was chosen prone, lateral or supine position. CT scan (PHILIPS 16-slice spiral CT, the Netherlands) was performed to locate the liver tumors. If necessary, contrast-enhanced CT was used to detect the boundary of tumor adjacent to vessels. According to the preoperative TPS, the puncture path was delineated on the CT scan images. Routine disinfection and local anesthesia were performed around the selected puncture points. The number and angle of the inserted 18G seed spinal needles were consistent with those of the preoperative TPS plan. The needle core was pulled out, and then, a ^125^I seed was released every 0.5 cm within the target lesion using a ^125^I seed implantation gun (Yunke Pharmaceuticals Limited Liability Company, Chengdu, China). Chest and abdominal CT scans were performed immediately after the procedure to assess the seed distribution and any complications and the CT scan images were imported into the TPS for postoperative dose verification. Patient follow-up.

The follow-up time was defined as the period from the start of ^125^I brachytherapy to the patient’s death or the last follow-up. Patients lost to follow-up were censored at the date of the last observation. Follow-up data included patient survival, laboratory data, and all follow-up treatment. According to the diagnostic criteria of AFP level for HCC, the AFP level of 400 ng/ml was defined as the cutoff value for the high and low AFP groups. In addition, contrast-enhanced CT and MR imaging of the abdomen was carried out to evaluate the local residual or recurrent tumor. The first period of follow-up assessment was performed one month after the ^125^I brachytherapy, and subsequent follow-up assessments were conducted every three months. Follow-up images were independently reviewed by one radiologist (> 10 years of experience) and one interventional physician (> 10 years of experience) in our center.

### Evaluation of tumor response and control

Efficacy assessment of tumor therapy was performed according to the Modified Response Evaluation Criteria (m-RECIST) in Solid Tumors [22]. Complete response (CR) was defined as the disappearance of arterial phase enhancement in the target lesion. Partial response (PR) was the size of the lesion decreased by more than 30% in arterial phase enhancement. Progressive disease (PD) was defined as ≥ 20% increase in the sum of diameters of target lesions (enhanced arterial phase). Stable disease (SD) was between PR and PD. The LPFS was defined as the period from the start of ^125^I brachytherapy to the date of the first imaging assessment of PD.

### Evaluation of complications

The perioperative complications were classified as major and minor, such as pain, fever, pneumothorax, or bleeding, and recorded in accordance with the Common Terminology Criteria for Adverse Events (CTCAE) Version 4.0.3 [23] and the Society of Interventional Radiology [24].

### Statistical analysis

All statistical analyses were performed using SPSS 26.0 (IBM, Chicago, Illinois, USA). Continuous variables were presented as the median and interquartile range (IQR), and categorical variables were described by frequency and percentage. The Cox model analyzed baseline tumor parameters, and the median time of LPFS was calculated using Kaplan–Meier curves and the log-rank tests. For all analyses, *p* values < 0.05 were considered statistically significant.

## Results

**Fig. 2:**
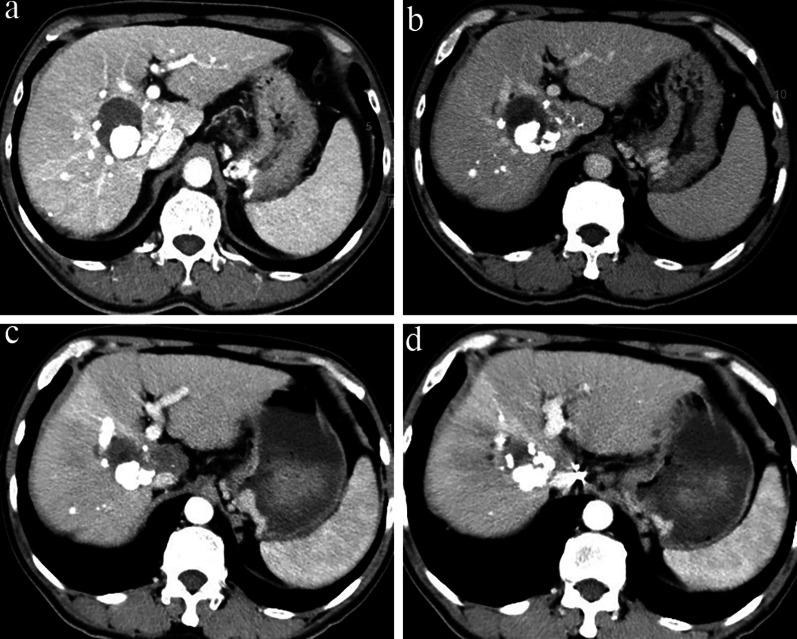
Contrast-enhanced computed tomography (CT) of a 69-year-old man with primary liver cancer (5.6 cm) adjacent to inferior vena cava and portal vein. Contrast-enhanced CT showed abnormal enhancement of liver S1 segment at 1 month after TACE (**a**). CT-guided MWA was performed but contrast-enhanced CT still showed residual tumor lesion (**b**) after 1 month. Three months after the CT-guided.^125^I seed implantation, the residual tumor lesions showed no enhancement (**c**, **d**)

The clinical characteristics of the patients are summarized in Table 1. A total of 114 HCC patients with residual or recurrent lesions after thermal ablation were included. The preoperative average prescribed dose D90 was 124.4 (90–131) Gy, and the mean V100 was 96.7% (84.8–99.9%). The average postoperative prescribed dose D90 was 122.7 (42–235) Gy, and the average V100 was 91.8% (72–100%). The median number of seeds was 20 (5–119). Representative CT images of HCC adjacent to inferior vena cava and portal vein before TACE, subsequent microwave ablation (MWA), and two months after the ^125^I brachytherapy are displayed (Fig. 2).

**Table 1:** Baseline characteristics of patients and tumors

Variables	Total (*n* = 114)
Age (years)	58 (26–84)
Sex	
Male	93 (82%)
Female	21 (18%)
Hospitalization frequency (times)	4 (1–21)
Diameter (cm)	
≥ 3	74 (65%)
< 3	40 (35%)
Number of intrahepatic tumors	
≥ 3	39 (31%)
< 3	75 (69%)
PVTT	
No	92 (81%)
Yes	22 (19%)
Adjacent organs	
Diaphragm	29 (26%)
Vessels	21 (18%)
Subcapsule	14 (12%)
No	50 (44%)
AFP	
< 400 ng/ml	98 (86%)
≥ 400 ng/ml	16 (14%)
Received resection before	
Yes	41 (36%)
No	73 (64%)
Received TACE before	
Yes	65 (57%)
No	49 (43%)

Continuous variables are expressed as the interquartile range (IQR); categorical variables are expressed as *n* (%)

*PVTT* portal vein tumor thrombus, *AFP* a-fetoprotein, *TACE* transcatheter arterial chemoembolization

**Fig. 3:**
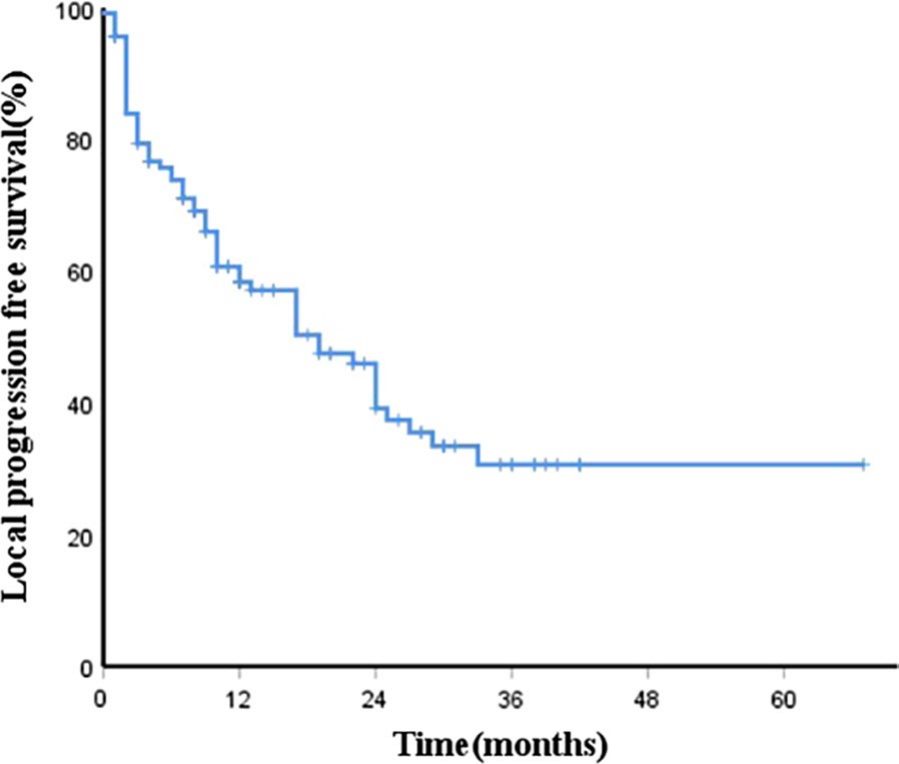
Kaplan–Meier curves of local tumor progression-free survival (LTPFS). The median LTPFS was 19.0 months. The 1-, 2-, and 3-year LTPFS rates were 58.7%, 50.0%, and 41.2%, respectively

**Fig. 4:**
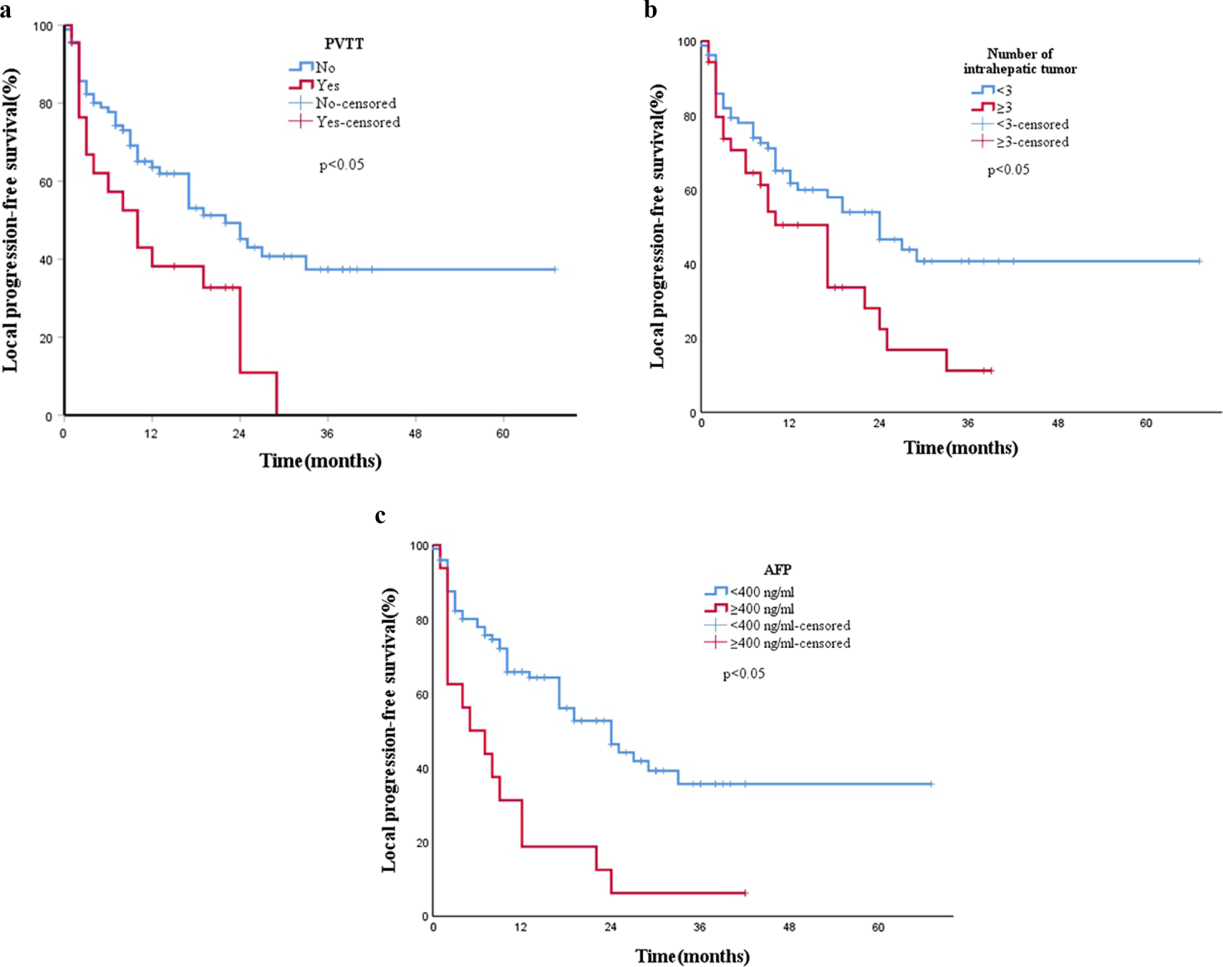
**a** PVTT VS no PVTT (*p* = 0.03),** b** number of intrahepatic tumors: < 3 VS ≥ 3 (*p* = 0.01), **c** AFP level: < 400 ng/ml VS ≥ 400 ng/ml (*p* = 0.02)

The CR, PR, and SD rates 6 months after the ^125^I brachytherapy procedure were 62.2%, 16.7%, and 7.9%, respectively. The form of tumor progression was mainly a local uncontrolled tumor or new lesions occur near the primary site. The median LPFS was 19.0 months. The 1-, 2-, and 3-year LPFS rates were 58.7%, 50.0%, and 41.2%, respectively (Fig. 3). Univariate analyses showed (Table 2) that PVTT, number of intrahepatic tumors, and AFP level were significantly associated with worse LPFS (*p* < 0.05). Multivariate analysis demonstrated (Table 3) that PVTT (hazard ratio [HR] = 2.04 [95% confidence interval (CI) 1.08–3.85]; *p* = 0.03), the number of intrahepatic tumors (HR = 2.50 [95% (CI) 1.43–4.35]; *p* = 0.01) and AFP level (HR = 2.36 [95% CI: 1.14–4.85]; *p* = 0.02) were independent risk factors for poor prognosis (Fig. 4). The median LPFS in patients without PVTT (25.2 months) was much longer compared to those with PVTT (9.9 months). The median LPFS in patients with less than three intrahepatic lesions was improved from 12.7 months to 28.3 months. The median LPFS was only 7.5 months in the high AFP group, but was prolonged with the decrease in AFP level (23.6 months).

**Table 2:** Univariate analysis of the risk factors of local progression-free survival

Factor	Number	*p* value
Sex		0.4
Male	93	
Female	21	
Hospitalization frequency		0.78
≥ 3	75	
< 3	39	
PVTT		0.01
No	92	
Yes	22	
Adjacent organs		0.72
Yes	64	
No	50	
AFP		0.001
< 400 ng/ml	98	
≥ 400 ng/ml	16	
Received resection before		0.61
Yes	41	
No	73	
Received TACE before		0.8
Yes	65	
No	49	
Number of intrahepatic tumors		0.01
≥ 3	39	
< 3	75	
Diameter (cm)		0.08
≥ 3	74	
< 3	40	

*PVTT* portal vein tumor thrombus, *AFP* a-fetoprotein, *TACE* transcatheter arterial chemoembolization

*p* values < 0.05 were considered to be statistically significant

**Table 3:** Multivariate analysis of the risk factors of local tumor progression-free survival

Factor	Hazard ratio	95% CI	*p* value
PVTT			0.03
No			
Yes	2.04	1.08–3.85	
Number of intrahepatic tumors			0.01
< 3			
≥ 3	2.50	1.43–4.35	
AFP			0.02
< 400 ng/ml			
≥ 400 ng/ml	2.36	1.14–4.85	
Diameter (cm)			0.78
< 3			
≥ 3	1.48	0.86–2.55	

*AFP* a-fetoprotein, *PVTT* portal vein tumor thrombus, *95% CI* 95% confidence intervals

*p* values < 0.05 were considered to be statistically significant

### Complications

No severe complications were detected during the perioperative period. Fourteen patients developed postoperative pain controlled with opioids. Five patients developed self-limiting pneumothorax. No radiation-related hepatitis was found in laboratory tests, and no ^125^I seeds migrated to other tissues or organs.

## Discussion

Since most patients with HCC are diagnosed in advanced stages, only about 20% of patients have the opportunity for surgery [25]. Local ablation therapy has been widely used in recent years and has provided curative outcomes for patients with early-stage liver cancer. However, previous studies showed that in patients with unresectable or special location of hepatocellular carcinoma, tumors have a risk of incomplete ablation or major complications after thermal ablation [26–28]. Subsequent ^125^I brachytherapy can improve the local tumor control rate for patients with local recurrent or residual lesions after thermal ablation. In our study, after the postoperative half-year the percentage of patients who achieved CR, PR, and SD was 57%, 13.2%, and 5.2%, respectively. The median LPFS was 19.0 months. The 1-, 2-, and 3-year PFS rates were 58.7%, 50.0%, and 41.2%, respectively.

Thermal ablation is comparable to radical resection for HCC patients with a diameter of ≤ 3 cm [6–8]. However, in previous studies, a diameter > 3 cm and a location adjacent to the major vessels, the diaphragm, or the subcapsular were considered major risk factors for local residual or recurrent HCC after thermal ablation [29, 30]. Previous studies have shown that a tumor size > 3 cm and a perivascular location result in a substantial reduction in the rate of tumor ablation [31]. A multipolar RFA system instead of a monopolar system, overlapping ablation, temporary reduction of blood flow by transient vascular occlusion or increased power output, and prolonged ablation time were applied to patients with these risk factors [32–34]. In the previous study of Peng et al. and Morimoto et al. [35], local ablation combined with TACE was applied in patients with isolated or multiple tumors 3–7 cm in diameter and improved overall survival (OS) and recurrence-free survival compared to the only RFA group. For local residual or recurrent lesions after ablation, TACE or repeat ablation was applied. In our study, ^125^I brachytherapy was performed in HCC patients, especially in those with tumors larger than 3 cm or that were adjacent to high-risk locations. A total of 40 patients with a tumor diameter > 3 cm and 42 with the tumor near high-risk locations achieved local CR. Univariate and multivariate analysis indicated that PVTT, the number of intrahepatic tumors, and the level of AFP were poor prognostic factors, whereas tumor size was not. However, previous studies have shown that the maximum diameter was a poor prognostic affecting LPFS [36]. The key difference between these studies and ours may be that we only enrolled patients with local residual or recurrent HCC after thermal ablation.

In certain locations (near major bile duct trees, abdominal organs, or heart), RFA or MWA is contraindicated due to the risk of serious complications and the heat sink effect, which results in a loss of efficacy. For subcapsular tumors, especially those protruding from the liver capsule, percutaneous puncture ablation may cause the risk of liver rupture and hemorrhage, or for liver cancer that is difficult to image-guided, laparoscopic or open surgical ablation can be considered [37]. When the tumor was adjacent to the important organs in abdominal organs, such as the diaphragm, stomach, bowel loops, and gallbladder, hydrodissection was performed before thermal ablation to reduce the thermal damage to the surrounding tissue and prevent major complications such as perforation. However, hydrodissection may lead to incomplete ablation and increase the risk of peritoneal seeding [38]. In our study, LPFS had no significant difference in the adjacent organ group compared to the control group after ^125^I seed implantation and suggesting that ^125^I brachytherapy is feasible and effective for local residual or recurrent lesions after thermal ablation [16].

Internal radiotherapy was divided into temporal high-dose-rate interstitial brachytherapy (HDR-BT) and permanent low-dose-rate interstitial brachytherapy (LDR-BT) according to the dose rate. Mohnike’s study suggested that CT-guided HDR-BT yielded a low rate of major complications and high one-year local recurrence-free surviving proportion [39]. HDR-BT combined with transarterial radioembolization (TARE) may have a positive effect on survival [40]. ^125^I brachytherapy is also used in HCC patients and improves OS and PFS. A systematic review and meta-analysis showed that TACE plus ^125^I brachytherapy had significantly improved the 6-month OS compared to TACE alone [41]. In our study, CR achieved 57% and the median LPFS prolonged up to 19.0 months. At present, there is no study comparing HDR with LDR in HCC. Thus, randomized clinical trials (RCT) comparing the two methods are needed.

^125^I brachytherapy is associated with only minor complications. Our previous study reported no deaths or severe complications in patients who underwent ^125^1 brachytherapy [21]. Although 17 cases appeared with minor complications (such as fever and pain) and five patients developed self-limiting pneumothorax in our study, all recovered after symptomatic treatment. Thus, CT-guided ^125^I brachytherapy was a safe method and another choice for treating local residual or recurrent tumors after thermal ablation with its curative effect, minimal surgical trauma, and few complications.

The results of our study suggest that ^125^I brachytherapy combined thermal ablation may be an effective and safe treatment mode for locally advanced HCC patients, especially with larger than 3 cm or adjacent to abdominal organs, and could inhibit tumor growth through different mechanisms and increase the local control rate. For locally advanced HCC, the treatment mode is mostly systemic drugs combined with local therapy which may be the future development direction, for example, sorafenib combined with TACE [42]. However, our study has several limitations that warrant discussion. First, the study was retrospective in nature, and the data were collected from a single institution. Second, during the follow-up period, some patients who experienced local progressive disease received subsequent treatments such as targeted therapy and immunotherapy but were not further analyzed. Third, this study mainly analyzed the effect of ^125^1 seed implantation combined with thermal ablation on LTPFS but not analyzed on OS or PFS. Therefore, randomized controlled trials are needed to support our findings of ^125^I brachytherapy for local residual or recurrent HCC after thermal ablation.

## Data Availability

The datasets used or analyzed during the current study are available from the corresponding author on reasonable request.

## Abbreviations

AFP: A-fetoprotein
CR: Complete response
CT: Computerized tomography
CTV: Clinical target volume
DVH: Dose-volume histogram
HCC: Hepatocellular carcinoma
LPFS: Local progression-free survival
LTP: Local tumor progression
MRI: Magnetic resonance imaging
MWA: Microwave ablation
PD: Progressive disease
PR: Partial response
PTV: Planned target volume
PVTT: Portal vein tumor thrombus
RFA: Radiofrequency ablation
TACE: Transarterial chemoembolization
SD: Stable disease
TPS: Treatment planning system
